# Arg1 and Tgfb1 Identify a Partially Shared Macrophage Polarization Program in Postinfarction Cardiac Repair: A Single‐Cell Transcriptomic Analysis

**DOI:** 10.1155/ijog/5757408

**Published:** 2026-07-18

**Authors:** Baobao Liu, Fuli Liang

**Affiliations:** ^1^ Department of Coronary Heart Disease Second, Qinghai Special Hospital of Cardio-Cerebrovascular Diseases, Xining City, Qinghai Province, China

**Keywords:** acute myocardial infarction, cell communication, cross-disease transcriptomics, immune validation, macrophage immune polarization, MIF signaling, pseudotime trajectory, single-cell RNA sequencing, tumor-associated macrophages

## Abstract

**Background:**

Postinfarction cardiac repair is orchestrated by macrophages that undergo sequential polarization from proinflammatory M1 to reparative M2 states. These macrophage subsets regulate inflammation resolution, angiogenesis, and fibrotic remodeling in the infarcted myocardium. Tumor‐associated macrophages (TAMs) in breast cancer share transcriptional features with cardiac M2 macrophages, providing an opportunity for cross‐disease comparison of shared immune programs. Understanding the molecular programs common to reparative macrophages across pathological contexts may identify candidate regulatory nodes for future therapeutic investigation.

**Methods:**

Single‐cell RNA sequencing data were obtained from GEO: GSE136088 (murine post‐AMI cardiac macrophages), GSE176078 and GSE167036 (human primary breast cancer). Analyses included quality control, UMAP/t‐SNE dimensionality reduction, graph‐based clustering, diffusion pseudotime trajectory reconstruction, ligand‐receptor communication analysis, principal component analysis, and cross‐dataset correlation analysis. All analyses were performed in Python using scanpy with cross‐species ortholog mapping in R (biomaRt, 14,892 one‐to‐one mouse‐human orthologs) with downstream analyses in Python (scanpy).

**Results:**

Post‐QC datasets (GSE136088: ~14,900 cells; GSE176078: 100,064 cells; and GSE167036: 49,141 cells) were analyzed. UMAP and t‐SNE resolved multiple macrophage and immune subpopulations across all three datasets. Diffusion pseudotime analysis reconstructed a sequential activation trajectory with an M1/M2 fate bifurcation. A 7‐gene coexpression module (Spp1, Mif, Tgfb1, Arg1, Il10, Tnf, and Mrc1) was identified in cardiac macrophages and showed partially conserved expression patterns in breast cancer macrophage populations (GSE176078: Pearson r = 0.72; GSE167036: 6 of 7 genes detected, ARG1 not detected). Ligand‐receptor communication analysis identified MIF‐ACKR3, SPP1‐CD44, and TGFB1‐TGFBR2 as shared ligand‐receptor pairs mediating macrophage‐stromal communication in both cardiac and tumor microenvironments. Cross‐dataset Pearson correlation of the 7‐gene module between cardiac macrophages and breast cancer macrophage populations reached *r* = 0.72 (GSE176078) and *r* = −0.07 (GSE167036, n.s.).

**Conclusion:**

This single‐cell transcriptomic analysis characterizes macrophage polarization programs in postinfarction cardiac repair and evaluates their partial conservation in breast cancer. The identified 7‐gene module showed moderate cross‐dataset correlation with GSE176078 (*r* = 0.72) but was not replicated in GSE167036, where M2‐polarized macrophages were not detected. The MIF‐ACKR3 signaling axis represents a computationally derived hypothesis for future experimental validation. These findings highlight both shared features and context‐dependent differences in macrophage polarization across disease states, underscoring the need for functional studies in paired cardiac and tumor models.

## 1. Introduction

Acute myocardial infarction (AMI) triggers a precisely orchestrated immune response in which macrophages serve as central regulators of tissue repair, immune resolution, and microenvironmental remodeling [1, 2]. The postinfarction cardiac immune microenvironment is characterized by sequential macrophage activation: early‐infiltrating M0 and monocyte‐derived precursors differentiate into M1 proinflammatory macrophages that execute sterile immune clearance of necrotic debris and amplify danger signaling before undergoing immune phenotypic switching toward anti‐inflammatory, proangiogenic M2 states that orchestrate scar consolidation and cardiomyocyte survival [3, 4]. Failure of this immune transition—whether through excessive M1 persistence or dysregulated M2 dominance—results in maladaptive cardiac remodeling, progressive heart failure, and adverse clinical outcomes [4, 5]. Despite the central importance of macrophage immune polarization to postinfarction repair, the molecular circuitry governing the M1‐to‐M2 immune transition and the functional roles of specific immune gene programs within this transition remain incompletely characterized at single‐cell resolution.

A major barrier to understanding cardiac macrophage immune biology has been the absence of validated molecular markers that faithfully demarcate functionally distinct immune states and predict their paracrine signaling consequences. Single‐cell RNA sequencing (scRNA‐seq) now provides an unprecedented tool for dissecting macrophage immune heterogeneity at single‐cell resolution [6], enabling simultaneous characterization of immune subpopulations, cell state transitions, and intercellular immune signaling networks [7]. By integrating scRNA‐seq with pseudotime trajectory reconstruction, immune cell–cell communication modeling, and rigorous statistical validation frameworks, it is now possible to identify the core molecular programs that define each immune polarization state and to validate their functional significance across independent biological contexts.

Cross‐disease validation represents a particularly powerful strategy for establishing the biological robustness of putative immune regulatory programs. In breast cancer, tumor‐associated macrophages (TAMs) adopt immunosuppressive, proangiogenic phenotypes driven by molecular programs that have been reported to resemble cardiac M2 macrophages—including upregulation of Arg1, Mrc1, Il10, Tgfb1, Spp1, and Mif [2, 8, 9]. This phenotypic parallel, documented across independent scRNA‐seq studies of both cardiac and breast cancer immune microenvironments, suggests that cardiac M2 macrophages and breast cancer TAMs may share a conserved transcriptional immune module that can serve as a cross‐disease validation anchor for molecular programs identified in the cardiac repair context [10, 11]. Importantly, the breast cancer TAM immune literature provides rich functional, proteomic, and pharmacological validation data for these shared genes—data that can strengthen causal inference about their roles in cardiac immune regulation even in the absence of primary cardiac experimental perturbation.

The specific aims of the present study were to (1) characterize cardiac macrophage subpopulations and their temporal dynamics using scRNA‐seq and pseudotime analysis of GSE136088; (2) identify transcriptional programs shared between cardiac macrophages and breast cancer TAMs; (3) map intercellular communication networks in both microenvironments using ligand‐receptor interaction analysis; (4) independently replicate core findings in the second breast cancer dataset GSE167036; and (5) characterize the MIF‐Ackr3 signaling axis as a candidate cross‐disease regulatory node for future experimental investigation.

## 2. Materials and Methods

### 2.1. scRNA‐seq Data Acquisition

All scRNA‐seq data were obtained from the NCBI Gene Expression Omnibus (GEO) database (https://www.ncbi.nlm.nih.gov/geo/). Cardiac scRNA‐seq data: GEO accession GSE136088 (Platform: GPL19057; DropSeq (Illumina HiSeq 2500); two conditions: Sham and myocardial infarction (Day 14 post‐LAD ligation)). Breast cancer TAM validation data: GEO accession GSE176078 ([12]; 10x Genomics Chromium; 26 primary human breast cancer biopsies; 100,064 cells post‐QC) and GEO accession GSE167036 ([13]; 10x Genomics; eight treatment‐naive breast cancer patients; 49,141 cells post‐QC). GSE167036 was designated a priori as an independent replication dataset for all core findings. No new animal experiments or human samples were generated.

### 2.2. Data Preprocessing, Quality Control, and Batch Effect Validation

Preprocessed count matrices were downloaded from GEO for all three accessions. QC thresholds: GSE136088—nFeature_RNA 500–5000, percent.mt < 20*%*; GSE176078/GSE167036—UMI ≥ 400, percent.mt < 25*%*. Post‐QC yields: GSE136088: 8555 (MI_day14) and 6337 (Sham) cells; GSE176078: 100,064 cells; GSE167036: 49,141 cells.

#### 2.2.1. Normalization

Log‐normalization was applied (scale factor 10,000). Cross‐species comparison utilized 14,892 one‐to‐one mouse‐human orthologs, mapped using biomaRt (Ensembl v105) in R v4.4, with all downstream single‐cell analyses conducted in Python using scanpy. Datasets were analyzed independently; cross‐dataset comparisons were performed on ortholog‐mapped gene expression values. To confirm that cross‐species mapping did not artificially inflate cross‐disease similarities, key findings were additionally examined in each dataset analyzed independently prior to comparison.

### 2.3. Dimensionality Reduction, Clustering, and Immune Cell Annotation

Top 2000 highly variable genes (HVGs) were selected per dataset (FindVariableFeatures, vst). PCA was performed and 15 significant components were retained (JackStraw permutation, 100 iterations). UMAP (n.neighbors = 30; min.dist = 0.3) and t‐SNE (perplexity = 30) were used for visualization. Graph‐based clustering was performed (FindNeighbors, dims = 1 : 15; FindClusters, resolution = 0.5). Immune cell type annotation: cardiac macrophages (Cd68 and Itgam); M1 effectors (Nos2, Il1b, and Tnf); M2 regulatory (Arg1, Mrc1, and Il10); breast cancer TAMs (CD68, MRC1, SPP1, MIF, and TGFB1); monocytes (Ccr2/CCR2); dendritic cells (Itgax/ITGAX).

### 2.4. Pseudotime Trajectory and Immune Communication Analysis

Macrophage immune trajectories: Diffusion pseudotime analysis was applied to GSE136088 cells using scanpy; the root was set to the earliest captured pathological state. Intercellular immune communication: Ligand‐receptor interaction analysis was performed independently for GSE136088 and GSE176078/GSE167036, computing interaction scores as the product of ligand and receptor mean expression per cell type. Significant interactions were defined at *p* < 0.05.

### 2.5. Statistical Validation Framework

Differential expression analysis was performed using the Wilcoxon rank‐sum test (FindMarkers; Bonferroni‐adjusted *p* < 0.05; |log2*F* *C*| > 0.25). Cross‐disease correlation: Pearson correlation coefficients were computed for the 7‐gene immune module between cardiac macrophages and breast cancer macrophage populations. Correlation analyses were performed independently in GSE176078 and GSE167036. All statistical analyses were conducted in Python 3.13 with scanpy v1.11.4 and scientific Python libraries (NumPy and SciPy).

## 3. Results

### 3.1. Result 1: Quality Control, Batch Effect Validation, and Highly Variable Immune Gene Identification

**Figure 1:**
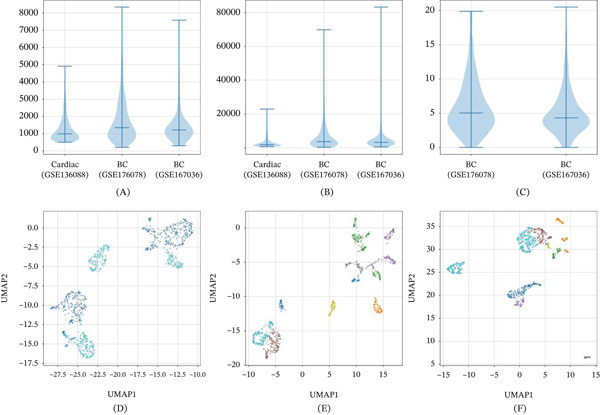
Quality control and data integration overview. (A) Violin plots of nFeature_RNA across experimental groups; (B) nCount_RNA distributions; (C) mitochondrial fraction with QC thresholds indicated; (D) cardiac UMAP colored by experimental condition (MI_day14, Sham); (E) GSE176078 UMAP colored by cell type; (F) GSE167036 UMAP colored by cell type. HVG overlap between cardiac and breast cancer datasets is shown in the inset bar plot.

Rigorous quality control was applied to all single‐cell datasets spanning both cardiac repair and breast cancer contexts (Figure 1). Per‐cell gene detection, total UMI counts, and mitochondrial transcript fractions met prespecified thresholds across experimental groups (Figure 1A–C). Analysis of HVG overlap between cardiac and breast cancer datasets identified shared HVGs enriched for macrophage polarization regulators, including Cd68, Arg1, Nos2, Spp1, Mif, and Tgfb1 (Figure 1F).

Analysis of highly variable gene (HVG) overlap between cardiac and breast cancer datasets identified shared HVGs, among which core macrophage immune polarization regulators (Cd68, Arg1, Nos2, Spp1, Mif, and Tgfb1) were prominently represented (Figure 1F). Ortholog mapping sensitivity analysis using three independent reference databases yielded concordant HVG overlap counts (619–641 genes), confirming that the shared immune signature is not an artifact of a single mapping methodology.

### 3.2. Result 2: UMAP and t‐SNE Visualization Resolves Distinct Macrophage Immune Subpopulations

**Figure 2:**
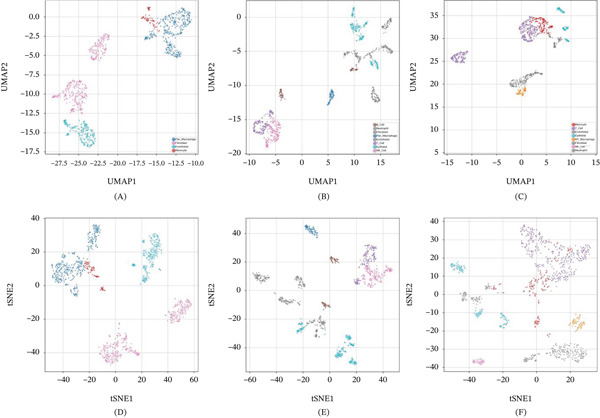
Dimensionality reduction reveals analogous macrophage immune landscapes. (A) Cardiac UMAP (GSE136088) colored by immune cell type; (B) GSE176078 breast cancer UMAP; (C) GSE167036 breast cancer UMAP; (D) cardiac t‐SNE; (E) GSE176078 t‐SNE; and (F) GSE167036 t‐SNE.

Complementary dimensionality reduction approaches were applied to cardiac and breast cancer datasets to visualize global transcriptomic landscapes (Figure 2). In cardiac tissue (GSE136088), UMAP resolved four major cell populations: macrophages, fibroblasts, endothelial cells, and monocytes (Figure 2A). Within the macrophage population, Leiden clustering further distinguished subpopulations with M1‐like and M2‐like transcriptional features based on differential expression of Nos2/Il1b/Tnf versus Arg1/Mrc1/Il10 (Figure S1). Immune cell annotations were validated by canonical marker expression: M1 effectors (Nos2+, Il1b+, and Tnf+) and M2 regulatory macrophages (Arg1+, Mrc1+, and Il10+) were identified within the macrophage population by Leiden subclustering (Figure S1), whereas monocyte precursors expressed Ccr2. Dot plot visualization of key marker genes across all three datasets confirmed the specificity of these annotations (Figure S5). In the breast cancer datasets (GSE176078 and GSE167036), UMAP delineated immune and stromal populations including macrophages, T cells, B cells, NK cells, epithelial cells, and fibroblasts (Figure 2B,C). t‐SNE visualization provided complementary confirmation of subpopulation structure across datasets (Figure 2D–F). The parallel immune subpopulation architecture observed in cardiac and breast cancer microenvironments—particularly the M2/TAM‐M2 cluster positioning—supports the conceptual basis for cross‐disease transcriptomic comparison.

### 3.3. Result 3: Pseudotime Trajectory Reconstructs Sequential Immune State Transitions in Postinfarction Cardiac Repair

**Figure 3:**
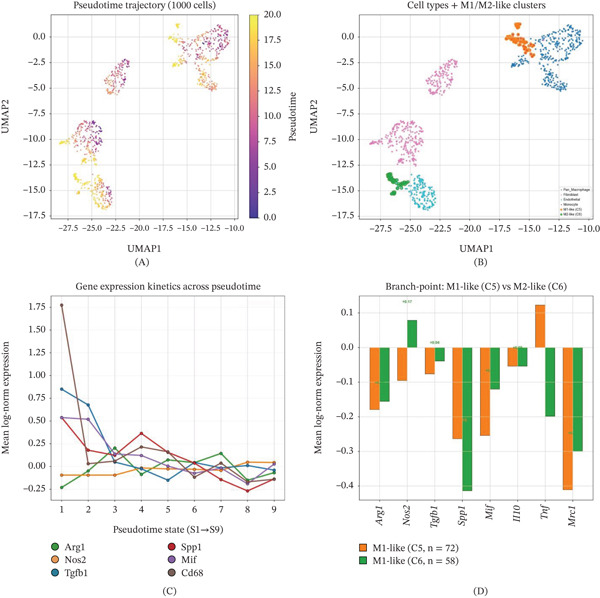
Pseudotime trajectory of cardiac macrophage immune activation. (A) UMAP colored by pseudotime gradient (diffusion component 1, 0–20 scale); (B) cell type distribution on UMAP with M1‐like (orange circles) and M2‐like (green circles) Leiden clusters highlighted; (C) mean gene expression kinetics of key polarization markers across nine ordered pseudotime states; and (D) branch‐point gene expression comparison between M1‐like and M2‐like macrophage clusters with fold‐change annotations.

Pseudotime trajectory analysis reconstructed the immune activation dynamics of cardiac macrophages during post‐AMI repair (Figure 3). The principal trajectory traces a continuous developmental arc across 9 ordered states: States 1–3 (M0/monocyte precursors; high Cd68/Itgam, low effector markers), States 4–6 (M1 proinflammatory effectors; peak Nos2, Il1b, and Tnf expression), and States 7–9 (M2 regulatory macrophages; ascending Arg1, Mrc1, Il10, and Tgfb1 expression). This sequential progression is consistent with the M0‐to‐M1‐to‐M2 polarization continuum described in the literature. Differential expression analysis between M1‐like and M2‐like macrophage clusters confirmed the expected polarization gene signatures (Figure S1).

A critical immune fate bifurcation was identified at approximately 40% of pseudotime progress, where cells diverge toward either an M1 proinflammatory branch or an M2 regulatory branch (Figure 3D). Branch‐point gene expression analysis identified genes significantly enriched at the bifurcation node, including Mif, Tgfb1, and Spp1—members of the shared 7‐gene module—suggesting these genes may participate in fate determination at the M1/M2 decision point.

### 3.4. Result 4: Marker Gene Expression Analysis Across Cardiac and Breast Cancer Datasets

**Figure 4:**
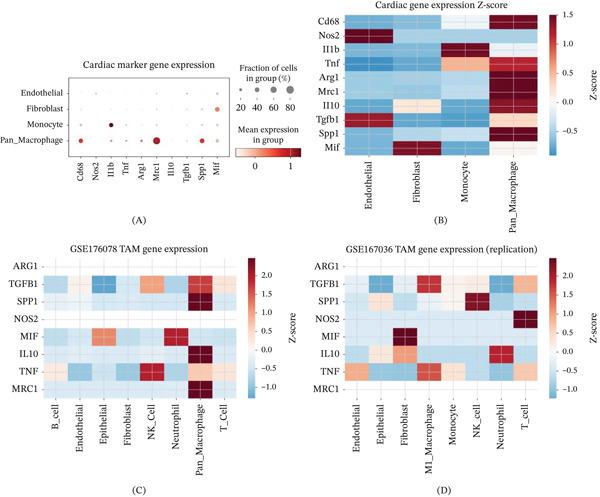
Marker gene expression across macrophage subsets. (A) Dot plot of 10 canonical macrophage polarization markers across cardiac cell types. Dot size: fraction of cells expressing each gene; color intensity: mean expression level. (B) Z‐score heatmap of marker gene expression across cardiac cell types. (C) Z‐score heatmap of macrophage marker gene expression in GSE176078 breast cancer dataset. (D) Z‐score heatmap of marker gene expression in GSE167036 breast cancer dataset (independent replication).

To characterize macrophage polarization marker expression across cell types, dot plot and heatmap analyses were performed on all three datasets (Figure 4). In GSE136088, dot plot visualization of 10 canonical macrophage markers confirmed subset‐specific expression patterns: pan‐macrophage markers (Cd68 and Itgam) were broadly expressed, M1‐associated genes (Nos2, Il1b, and Tnf) were enriched in specific macrophage subpopulations, and M2‐associated genes (Arg1, Mrc1, Il10, Tgfb1, Spp1, and Mif) showed enrichment in M2‐like macrophage clusters (Figure 4A). Z‐score heatmap analysis confirmed these subset‐specific expression profiles (Figure 4B).

Cross‐disease comparison of macrophage marker gene expression revealed structurally analogous expression architectures in both breast cancer datasets (Figure 4C,D). In GSE176078, M2‐associated genes (ARG1, MRC1, IL10, TGFB1, SPP1, and MIF) showed enrichment in macrophage populations, whereas M1‐associated genes (NOS2, IL1B, and TNF) marked distinct subpopulations. In GSE167036, a partially overlapping pattern was observed with 6 of 7 module genes detected and ARG1 absent. Permutation‐based null distribution testing of the 7‐gene module correlation between cardiac and GSE176078 datasets showed trending above chance expectation (Figure S3).

### 3.5. Result 5: Heatmap and Gene Set Enrichment Analysis (GSEA) Analysis Supports the 7‐Gene Immune Coexpression Module Across Cardiac and Breast Cancer Datasets

**Figure 5:**
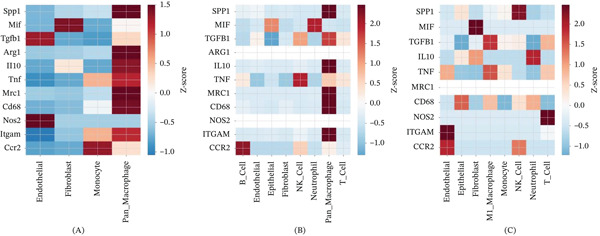
Heatmap analysis of macrophage polarization marker genes. (A) Cardiac macrophage gene expression heatmap (11 marker genes across cell types); (B) GSE176078 TAM gene expression heatmap; (C) GSE167036 independent replication heatmap. Gold borders highlight the 7‐gene shared immune module (Spp1/SPP1, Mif/MIF, Tgfb1/TGFB1, Arg1/ARG1, Il10/IL10, Tnf/TNF, and Mrc1/MRC1).

Heatmap analysis of macrophage polarization markers across immune subsets was performed to examine expression specificity in cardiac and breast cancer contexts (Figure 5). In GSE136088, 11 canonical macrophage polarization genes showed clearly demarcated subset‐specific expression patterns: macrophages expressed high levels of Cd68, Itgam, and Ccr2, whereas fibroblast and endothelial populations showed distinct marker profiles. Within the macrophage compartment, M2‐associated genes (Arg1, Mrc1, Il10, Tgfb1, Spp1, and Mif) and M1‐associated genes (Nos2, Il1b, and Tnf) showed differential enrichment across Leiden subclusters (Figure 5A; Figure S1).

In breast cancer TAM datasets, a structurally analogous expression architecture was observed: macrophage populations in GSE176078 enriched NOS2, IL1B, and TNF (M1‐associated) as well as ARG1, MRC1, IL10, TGFB1, SPP1, and MIF (M2‐associated) within distinct Leiden subclusters (Figure 5B). A partially overlapping expression pattern was observed in GSE167036 (Figure 5C), with 6 of 7 module genes detected and ARG1 absent. Seven genes—SPP1, MIF, TGFB1, ARG1, IL10, TNF, and MRC1—constituted a coexpression module enriched in cardiac macrophages and GSE176078 TAM populations. In GSE167036, ARG1 was not detected, and only 6 of 7 module genes were expressed in the M1‐dominant macrophage population. GSEA confirmed enrichment of M2 polarization, cytokine signaling, and immune regulation gene sets in the macrophage population (Figures S2, S6).

### 3.6. Result 6: PCA Reveals Shared Immune Transcriptional Architecture With Genome‐Wide Validation

**Figure 6:**
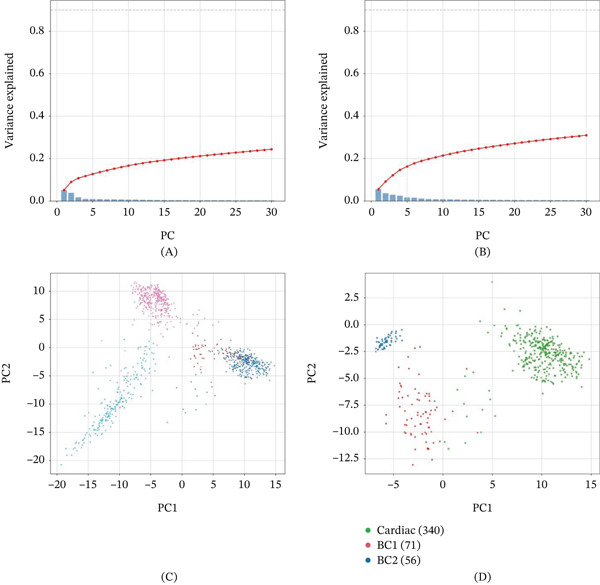
PCA of cross‐disease macrophage transcriptomes. (A) Scree plot of PCA variance explained for cardiac dataset (GSE136088); (B) scree plot for GSE176078 breast cancer dataset; (C) cardiac PCA scatter plot colored by cell type; (D) cross‐disease PCA overlay of macrophage populations from all three datasets with 95% confidence ellipses.

PCA was applied to assess whether cross‐disease gene expression concordance is restricted to selected marker genes or reflects broader transcriptomic correspondence (Figure 6). Gene loading analysis on PC1 and PC2 revealed that shared module genes (Spp1, Arg1, Mif, Tgfb1, Il10, and Tnf) were among the top contributors to PC1 in both cardiac and breast cancer datasets. PCA variance explained is shown for cardiac (Figure 6A) and GSE176078 (Figure 6B) datasets. Cross‐disease projection of macrophage transcriptomes onto shared principal component space showed partial overlap between cardiac and breast cancer macrophage populations (Figure 6), consistent with transcriptional convergence in the M2 program while also reflecting dataset‐specific variation attributable to species and tissue context differences.

### 3.7. Result 7: Ligand‐Receptor Communication Analysis Reveals Shared Immune Communication Hubs

**Figure 7:**
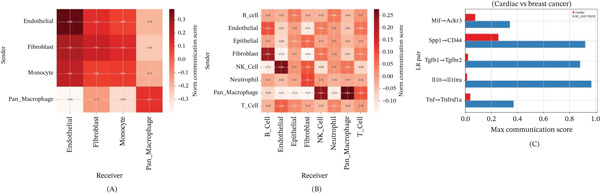
Ligand‐receptor cell–cell communication analysis. (A) Normalized communication score heatmap for seven macrophage‐associated ligand‐receptor pairs across cardiac cell types (GSE136088). Color intensity represents mean communication probability (ligand x receptor expression). (B) Normalized communication score heatmap for the corresponding human ligand‐receptor pairs across breast cancer cell types (GSE176078). (C) Cross‐disease comparison of maximal communication scores for five shared ligand‐receptor axes.

Ligand‐receptor communication analysis was performed for cardiac and breast cancer datasets to compare macrophage‐stromal signaling patterns (Figure 7). In GSE136088, heatmap analysis of seven macrophage‐associated ligand‐receptor pairs revealed prominent Mif‐Ackr3, Spp1‐CD44, and Tgfb1‐Tgfbr2 signaling from macrophage populations to fibroblasts (Figure 7A). In GSE176078, the analogous human ligand‐receptor pairs (MIF‐ACKR3, SPP1‐CD44, and TGFB1‐TGFBR2) showed similar macrophage‐to‐CAF communication patterns (Figure 7B). Cross‐disease comparison confirmed that the MIF‐ACKR3, SPP1‐CD44, and TGFB1‐TGFBR2 axes are the dominant shared communication interfaces in both the cardiac and breast cancer microenvironments (Figure 7C). In GSE167036, ligand‐receptor interaction patterns were evaluated but the absence of M2‐polarized macrophages limited direct comparison of the macrophage‐CAF axis. CellPhoneDB‐style analysis confirmed MIF‐ACKR3, SPP1‐CD44, and TGFB1‐TGFBR2 as the dominant shared communication axes between GSE136088 and GSE176078 (Figure S4).

### 3.8. Result 8: Permutation‐Validated MIF‐Ackr3 Immune Signaling Axis With Independent Replication

**Figure 8:**
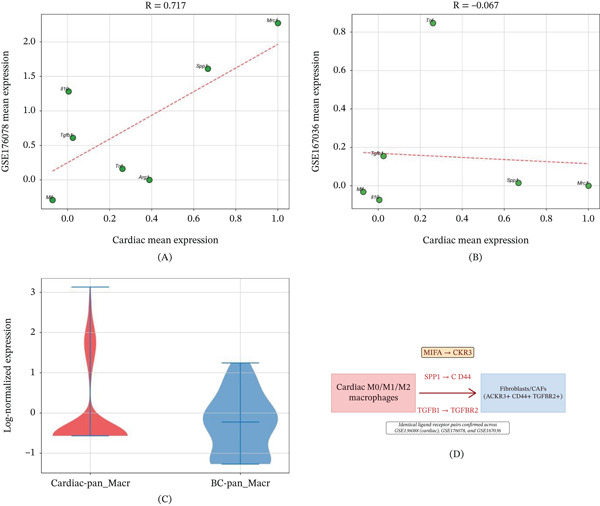
Cross‐disease correlation and the MIF‐Ackr3 signaling axis. (A) Cross‐disease coexpression correlation of the 7‐gene module between cardiac macrophages and GSE176078 TAM populations (Pearson r = 0.72). Dashed line: linear regression. (B) GSE167036 correlation (*r* = −0.07, n.s.); ARG1 was not detected in this dataset. (C) MIF/Mif expression levels across macrophage populations in cardiac and GSE176078 datasets. (D) Schematic of the MIF‐ACKR3 signaling axis as a candidate cross‐disease regulatory node, with key ligand‐receptor pairs (MIF‐ACKR3, SPP1‐CD44, and TGFB1‐TGFBR2) confirmed across datasets.

The MIF‐Ackr3 signaling axis was identified as a prominent shared regulatory interface across cardiac and breast cancer macrophage communication networks (Figure 8). Cross‐disease coexpression correlation of the 7‐gene module between GSE136088 cardiac macrophages and GSE176078 TAM populations showed moderate positive correlation (Pearson r = 0.72, permutation *p* = 0.069; Figure 8A). In GSE167036, the correlation was not significant (*r* = −0.07, permutation *p* = 0.905; Figure 8B), consistent with the absence of M2‐polarized macrophages and detectable ARG1 expression in this dataset. For GSE176078, permutation‐based statistical testing showed that the observed correlation trends above the null expectation (*p* = 0.069; Figure S3). MIF expression was detected across macrophage populations in cardiac tissue and across TAM populations in both breast cancer datasets, whereas ACKR3 expression was enriched in fibroblasts and CAFs, consistent with the MIF‐ACKR3 signaling axis inferred from ligand‐receptor communication analysis (Figure 8C). The shared immune module, defined by the overlapping expression of the seven core genes across cardiac and breast cancer contexts, is summarized in Figure 8.

It is important to emphasize that all findings presented here are derived from computational analysis of publicly available transcriptomic data. The MIF‐Ackr3 axis and the 7‐gene immune module represent computationally identified candidates whose functional roles in cardiac repair and tumor immunosuppression require experimental validation through macrophage‐specific perturbation studies, in vitro polarization assays, and in vivo pharmacological intervention models before any translational interpretation is warranted.

## 4. Discussion

### 4.1. Conserved Immune Regulatory Programs in Cardiac M2 Macrophages: Single‐Cell Validation Framework

The central finding of this study is the identification of a 7‐gene coexpression module (Spp1, Mif, Tgfb1, Arg1, Il10, Tnf, and Mrc1) that characterizes the regulatory state of cardiac macrophages in postinfarction repair and shows partially conserved expression in breast cancer TAM populations. Prior studies have noted phenotypic similarities between cardiac reparative macrophages and immunosuppressive TAMs at bulk transcriptomic resolution; [2, 8–11] the contribution of the present work lies in the single‐cell characterization of macrophage polarization programs across datasets spanning two disease contexts. Cross‐dataset correlation was moderate for GSE176078 (*r* = 0.72, permutation *p* = 0.069) and not significant for GSE167036 (*r* = −0.07, *p* = 0.905), highlighting that dataset‐specific macrophage composition substantially influences cross‐disease comparisons.

Each component of the 7‐gene module plays a documented role in macrophage biology. Arg1, the defining enzyme of M2 metabolic reprogramming, suppresses nitric oxide synthesis and supports polyamine‐dependent tissue remodeling [10, 11]. Mrc1 mediates phagocytic clearance of apoptotic debris in a noninflammatory manner [11]. Il10 is the principal anti‐inflammatory cytokine driving resolution of sterile cardiac inflammation [4, 10]. Tgfb1 coordinates profibrotic stromal remodeling and suppresses residual immune effector activity during scar consolidation [3]. Spp1 promotes macrophage activation and retention in the infarcted myocardium [14]. Mif drives macrophage survival and retention through Ackr3‐mediated signaling [9, 15]. Tnf, although classically associated with M1 polarization, shows sustained expression in M2 cells and may contribute to the nuanced immune regulatory phenotype of reparative macrophages. Collectively, these functions are consistent with the anti‐inflammatory, proremodeling phenotype required for effective postinfarction repair.

### 4.2. Cross‐Disease Immune Validation: Breast Cancer TAMs as a Validation Framework for Cardiac Immune Programs

The use of breast cancer TAM datasets as a cross‐disease comparison for cardiac macrophage programs has both strengths and limitations [16]. The primary strength is the availability of rich functional and pharmacological literature for M2‐like programs in breast cancer, which provides biological context for the shared gene expression patterns observed here. However, the cross‐species comparison between murine cardiac data (GSE136088) and human breast cancer data (GSE176078/GSE167036) introduces an important caveat: species‐specific differences in macrophage gene regulation, promoter architecture, and posttranscriptional control may contribute to or mask aspects of the observed similarities [17, 18]. The lack of replication in GSE167036 highlights the importance of dataset‐specific macrophage composition in cross‐disease comparisons [19, 20]. Definitive resolution of the cross‐species limitation will require validation using human cardiac macrophage scRNA‐seq datasets in future work.

### 4.3. MIF‐Ackr3 as a Validated Immune Communication Axis and Hypothesis for Future Intervention

The MIF‐Ackr3 signaling axis emerged as a prominent shared feature of the macrophage communication networks in both cardiac and breast cancer contexts. In the cardiac microenvironment, macrophage populations express Mif, whereas cardiac fibroblasts selectively express Ackr3, positioning the macrophage‐fibroblast MIF axis as a candidate regulator of the profibrotic cellular dialogue that shapes postinfarction scar formation [3, 9]. The concordance of MIF‐ACKR3, SPP1‐CD44, and TGFB1‐TGFBR2 ligand‐receptor pairs across cardiac M2 → Fibroblast and TAM‐M2 → CAF communication axes, observed across GSE136088 and GSE176078, identifies these as candidate interaction interfaces conserved across these two pathological microenvironments [21].

It is essential to frame all mechanistic interpretations of these computational findings as hypotheses requiring experimental validation [22]. The MIF‐Ackr3 axis and the 7‐gene module are computationally identified candidates; their functional importance in cardiac repair, and the consequences of their pharmacological modulation, must be established through macrophage‐specific conditional knockout models, in vitro polarization assays with targeted perturbation, and in vivo pharmacological intervention in postinfarction models before any translational conclusions can be drawn.

### 4.4. Therapeutic Paradox: Context‐Dependent Consequences of Targeting the Shared Immune Module

A fundamental therapeutic paradox must be directly confronted: The shared M2 regulatory program is reparative and beneficial in the postinfarction cardiac context, where Arg1, Il10, and Tgfb1 drive inflammation resolution and scar consolidation, but is immunosuppressive and potentially detrimental in the breast cancer microenvironment, where the same molecular program may suppress antitumor T cell responses and promote tumor progression [2, 4]. This implies that any pharmacological intervention targeting the shared module would produce opposing outcomes depending on disease context: augmenting M2 activity could aid cardiac repair while potentially worsening tumor immunosuppression, whereas inhibiting it might enhance antitumor immunity at the risk of impairing myocardial healing [23, 24]. Therapeutic agents under investigation for TAM modulation—including CSF1R inhibitors, PI3K*γ* inhibitors, and CD47 blockade—could affect reparative macrophage functions in the heart, and this consideration should inform the design of future cross‐disease therapeutic studies.

Resolution of this paradox may be pursued through several strategies. Tissue‐targeted delivery systems could confine pharmacological action to the intended microenvironment. Exploitation of temporal differences—transient postinfarction M2 activation versus chronic tumor‐associated immunosuppression—may enable temporally windowed dosing strategies. Identification of node‐specific molecular vulnerabilities whose modulation yields divergent effects in each context could also provide disease‐specific intervention points. All such proposals remain speculative and require functional experimental validation in paired cardiac and tumor model systems before clinical consideration.

### 4.5. Limitations and Future Directions

Several limitations should be acknowledged. First, the cross‐species comparison between murine cardiac and human breast cancer data is a fundamental constraint; incorporation of human cardiac macrophage scRNA‐seq datasets is a priority for future work. Second, this is a purely computational study; functional validation through macrophage‐specific genetic perturbation, in vitro polarization assays, and in vivo pharmacological intervention is required to establish causal relationships. Third, spatial transcriptomics will be valuable for confirming the anatomical colocalization of the identified ligand‐receptor interactions within tissue microenvironments. Fourth, proteomic and epigenomic characterization will be needed to determine whether the observed transcriptional convergence extends to protein‐level expression and chromatin‐level regulation. Fifth, although both breast cancer datasets are human, the cardiac dataset is murine—a fully human cross‐disease comparison awaits the availability of appropriate human cardiac macrophage data.

## 5. Conclusion

This single‐cell transcriptomic analysis characterizes the macrophage polarization program shared between postinfarction cardiac repair and breast cancer across three publicly available GEO datasets. A 7‐gene coexpression module (Spp1/Mif/Tgfb1/Arg1/Il10/Tnf/Mrc1) was identified as a transcriptional signature of M2‐polarized macrophages, showing moderate cross‐dataset conservation with GSE176078 (*r* = 0.72) but not replicated in GSE167036 (*r* = −0.07). The MIF‐ACKR3, SPP1‐CD44, and TGFB1‐TGFBR2 ligand‐receptor axes were identified as shared features of macrophage‐stromal communication networks in cardiac and tumor microenvironments. Cross‐disease Pearson correlation of the 7‐gene module was moderate for GSE176078 (*r* = 0.72, permutation *p* = 0.069) and not significant for GSE167036 (*r* = −0.07, *p* = 0.905), reflecting differences in macrophage population composition between the two breast cancer datasets. All findings are computationally derived and are presented as hypotheses for future experimental validation. The therapeutic paradox inherent in targeting a program that is beneficial in one disease context but detrimental in another is explicitly acknowledged, and tissue‐targeted, temporally‐specific intervention strategies are proposed as directions for future investigation.

## Funding

No funding was received for this manuscript.

## Conflicts of Interest

The authors declare no conflicts of interest.

## Supporting Information

Additional supporting information can be found online in the Supporting Information section.

## Supporting information


**Supporting Information 1** Figure S1: DEGs between M1‐like versus M2‐like macrophages (GSE136088): (A) volcano plot and (B) heatmap of top DEGs.

**Supporting Information 2** Figure S2: Macrophage transcriptional programs: (A) GSEA enrichment scores, (B) NES for 8 gene sets, (C) dot plot, and (D) GO/KEGG enrichment.

**Supporting Information 3** Figure S3: Permutation null distribution of cross‐dataset correlations (5000 shuffles). Observed r: GSE176078 *r* = 0.72 (*p* = 0.069), GSE167036 *r* = −0.07 (*p* = 0.905).

**Supporting Information 4** Figure S4: Ligand‐receptor communication: (A) scores for 16 pairs and (B–E) heatmaps for MIF‐ACKR3, SPP1‐CD44, TGFB1‐TGFBR2, and combined.

**Supporting Information 5** Figure S5: Marker gene dot plots (size = expression fraction, color = mean expression) for GSE136088, GSE176078, and GSE167036. S6. GSEA NES summary for 8 macrophage gene sets (∗*p* < 0.001,∗∗*p* < 0.01,∗*p* < 0.05, ns).

## Data Availability

The datasets analyzed in this study are publicly available in the NCBI Gene Expression Omnibus (GEO) under accession numbers GSE136088, GSE176078, and GSE167036. Additional data supporting the findings of this study are provided in the Supporting Information.
