# Supplementary figures and images for: Arg1 and Tgfb1 Identify a Partially Shared Macrophage Polarization Program in Postinfarction Cardiac Repair: A Single‐Cell Transcriptomic Analysis
Source: Int J Genomics. 2026 Jul 18;2026:5757408. doi: 10.1155/ijog/5757408 (PMC13379942; doi:10.1155/ijog/5757408)

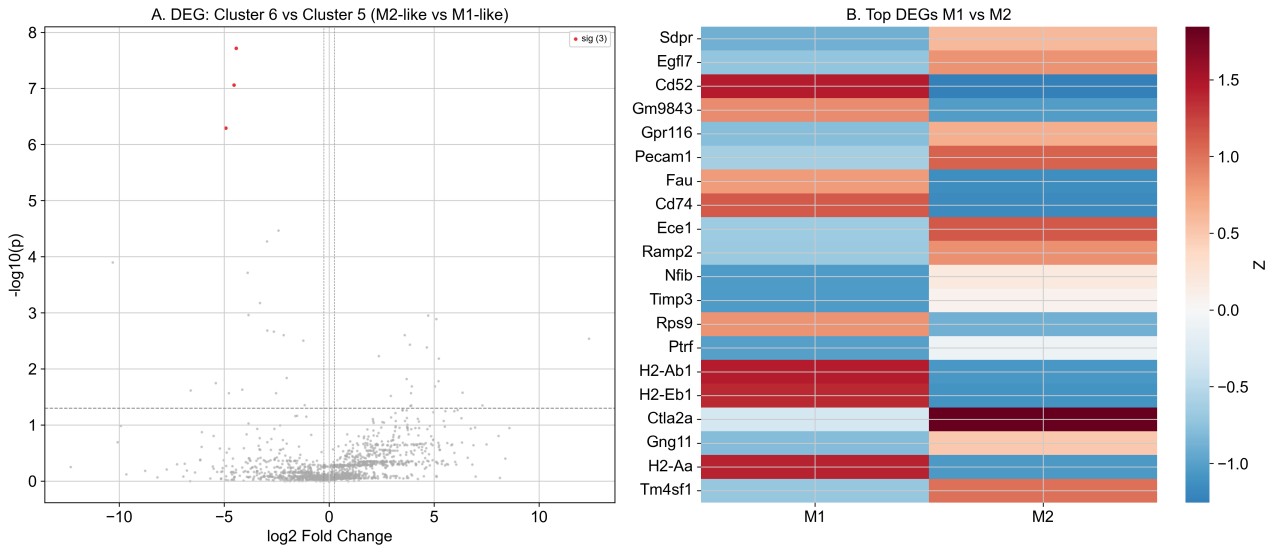

Supplement: Supplementary file 1 — Supporting Information 1 Figure S1: DEGs between M1‐like versus M2‐like macrophages (GSE136088): (A) volcano plot and (B) heatmap of top DEGs. [file IJOG-2026-5757408-s001.jpg]

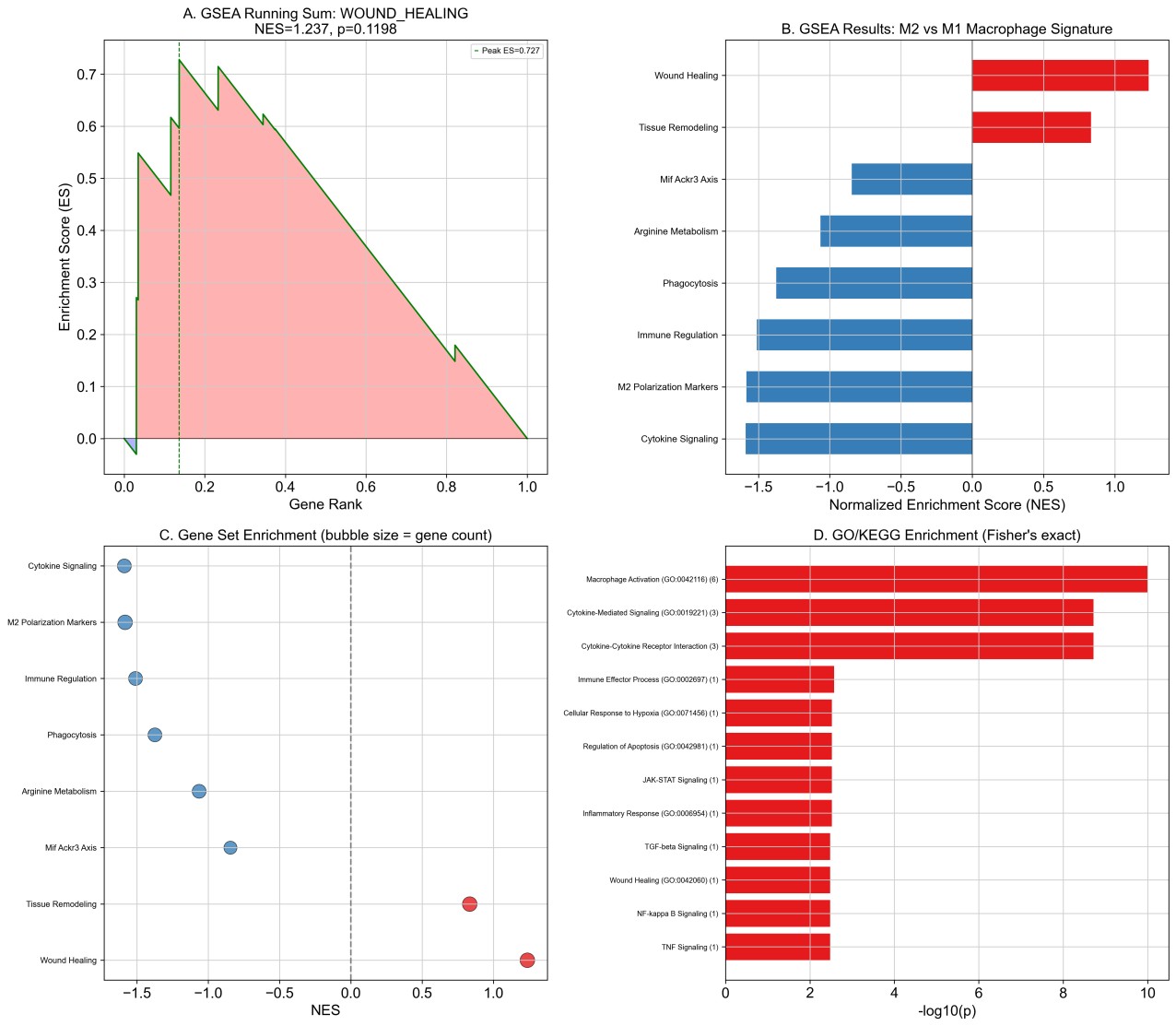

Supplement: Supplementary file 2 — Supporting Information 2 Figure S2: Macrophage transcriptional programs: (A) GSEA enrichment scores, (B) NES for 8 gene sets, (C) dot plot, and (D) GO/KEGG enrichment. [file IJOG-2026-5757408-s002.jpg]

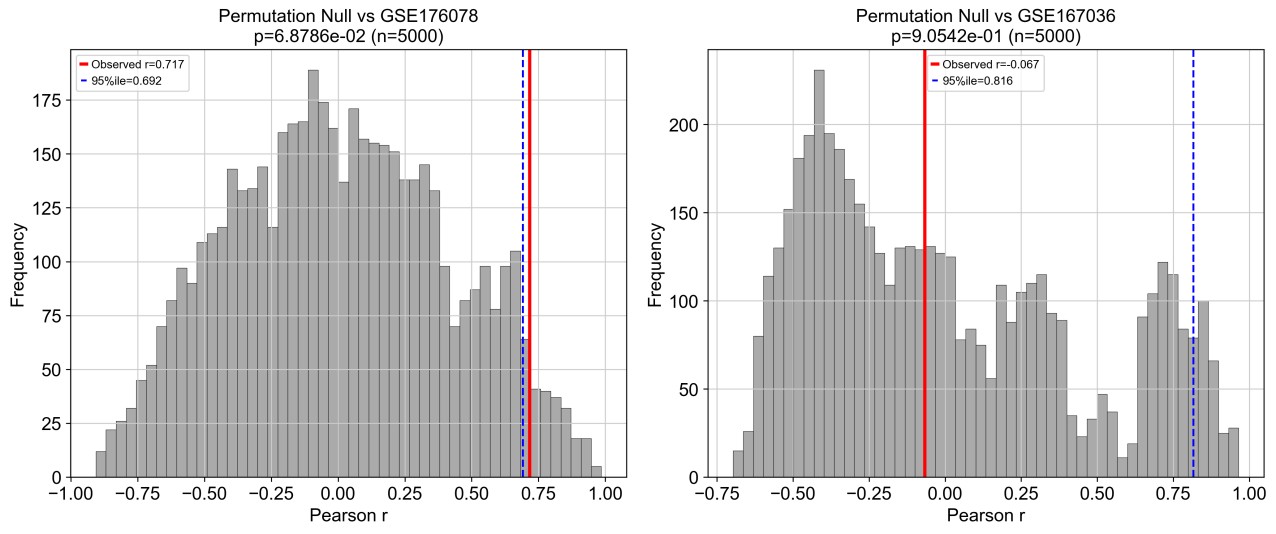

Supplement: Supplementary file 3 — Supporting Information 3 Figure S3: Permutation null distribution of cross‐dataset correlations (5000 shuffles). Observed r: GSE176078 r = 0.72 (p = 0.069), GSE167036 r = −0.07 (p = 0.905). [file IJOG-2026-5757408-s004.jpg]

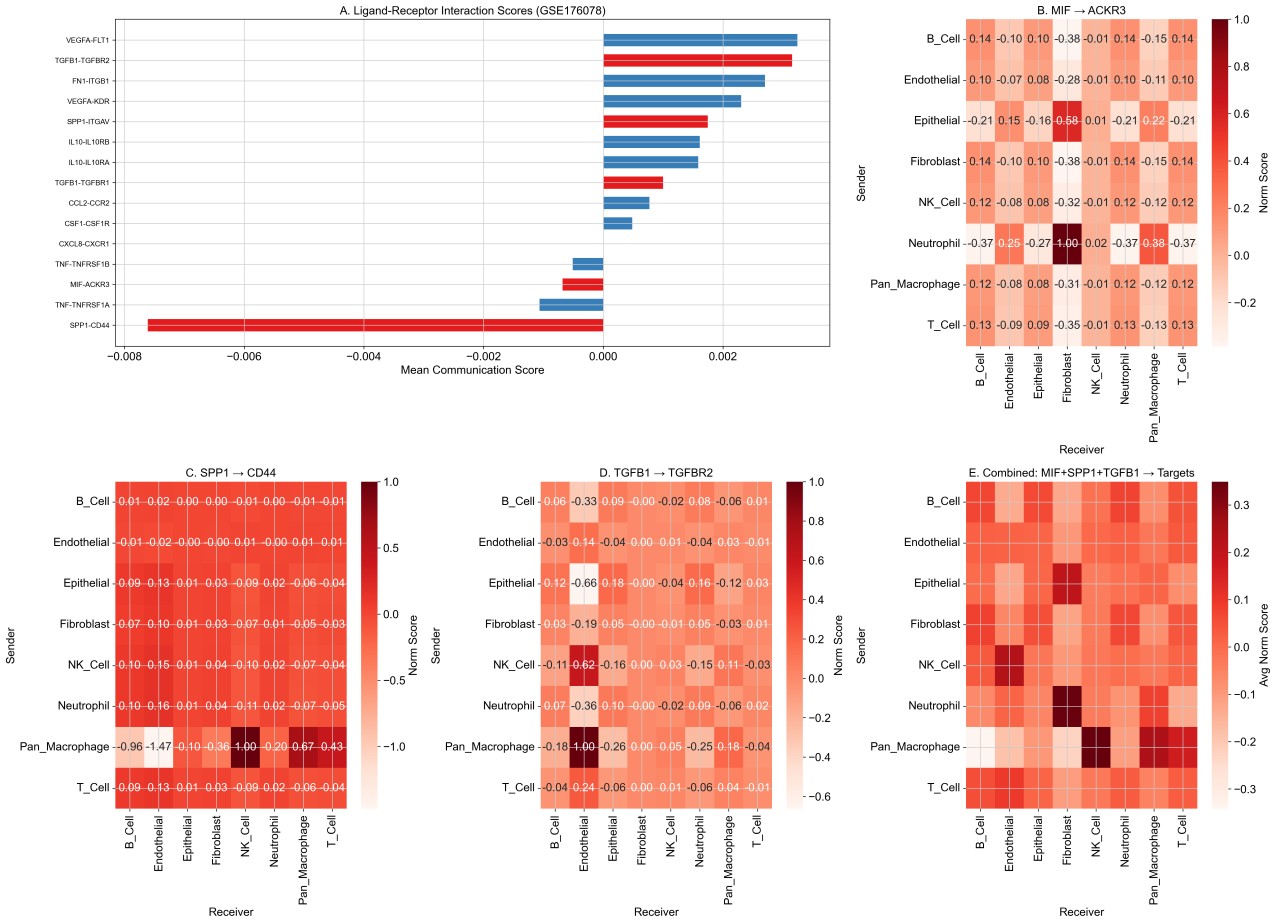

Supplement: Supplementary file 4 — Supporting Information 4 Figure S4: Ligand‐receptor communication: (A) scores for 16 pairs and (B–E) heatmaps for MIF‐ACKR3, SPP1‐CD44, TGFB1‐TGFBR2, and combined. [file IJOG-2026-5757408-s003.jpg]

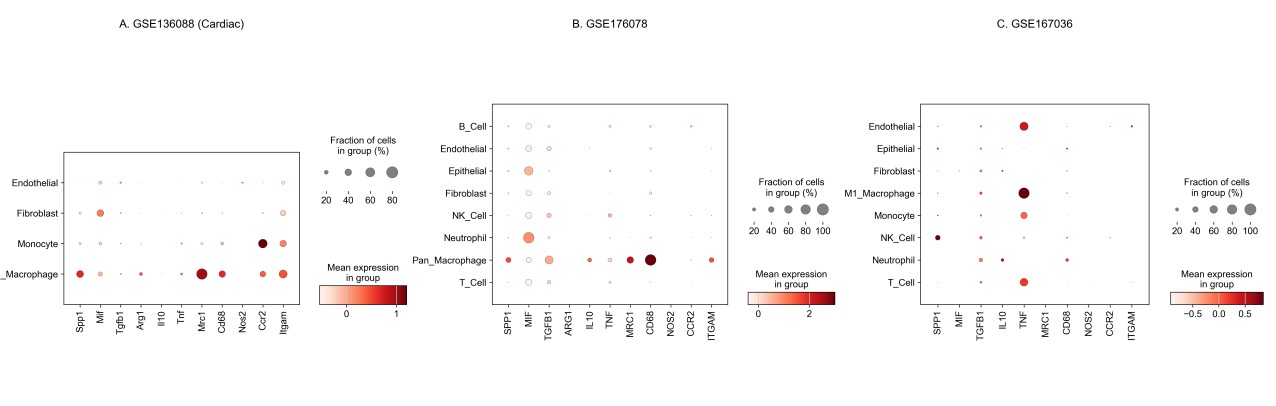

Supplement: Supplementary file 5 — Supporting Information 5 Figure S5: Marker gene dot plots (size = expression fraction, color = mean expression) for GSE136088, GSE176078, and GSE167036. S6. GSEA NES summary for 8 macrophage gene sets (∗p < 0.001,∗∗p < 0.01,∗p < 0.05, ns). [file IJOG-2026-5757408-s005.jpg]
